# Heme Mediated STAT3 Activation in Severe Malaria

**DOI:** 10.1371/journal.pone.0034280

**Published:** 2012-03-30

**Authors:** Mingli Liu, Audu S. Amodu, Sidney Pitts, John Patrickson, Jacqueline M. Hibbert, Monica Battle, Solomon F. Ofori-Acquah, Jonathan K. Stiles

**Affiliations:** 1 Department of Microbiology, Biochemistry and Immunology, Morehouse School of Medicine, Atlanta, Georgia, United States of America; 2 Department of Pathology, Morehouse School of Medicine, Atlanta, Georgia, United States of America; 3 Department of Pediatrics, Emory University School of Medicine, Atlanta, Georgia, United States of America; Institut National de la Santé et de la Recherche Médicale - Institut Cochin, France

## Abstract

**Background:**

The mortality of severe malaria [cerebral malaria (CM), severe malaria anemia (SMA), acute lung injury (ALI) and acute respiratory distress syndrome (ARDS)] remains high despite the availability associated with adequate treatments. Recent studies in our laboratory and others have revealed a hitherto unknown correlation between chemokine CXCL10/CXCR3, Heme/HO-1 and STAT3 and cerebral malaria severity and mortality. Although Heme/HO-1 and CXCL10/CXCR3 interactions are directly involved in the pathogenesis of CM and fatal disease, the mechanism dictating how Heme/HO-1 and CXCL10/CXCR3 are expressed and regulated under these conditions is still unknown. We therefore tested the hypothesis that these factors share common signaling pathways and may be mutually regulated.

**Methods:**

We first clarified the roles of Heme/HO-1, CXCL10/CXCR3 and STAT3 in CM pathogenesis utilizing a well established experimental cerebral malaria mouse (ECM, *P. berghei* ANKA) model. Then, we further determined the mechanisms how STAT3 regulates HO-1 and CXCL10 as well as mutual regulation among them in CRL-2581, a murine endothelial cell line.

**Results:**

The results demonstrate that (1) STAT3 is activated by *P. berghei* ANKA (PBA) infection *in vivo* and Heme *in vitro*. (2) Heme up-regulates HO-1 and CXCL10 production through STAT3 pathway, and regulates CXCL10 at the transcriptional level *in vitro*. (3) HO-1 transcription is positively regulated by CXCL10. (4) HO-1 regulates STAT3 signaling.

**Conclusion:**

Our data indicate that Heme/HO-1, CXCL10/CXCR3 and STAT3 molecules as well as related signaling pathways play very important roles in the pathogenesis of severe malaria. We conclude that these factors are mutually regulated and provide new opportunities to develop potential novel therapeutic targets that could be used to supplement traditional prophylactics and treatments for malaria and improve clinical outcomes while reducing malaria mortality. Our ultimate goal is to develop novel therapies targeting Heme or CXCL10-related biological signaling molecules associated with development of fatal malaria.

## Introduction

Malaria (*Plasmodium. falciparum*) infects 200 to 300 million people globally and kills approximately 900,000 (mostly children) every year. Current anti-malaria drugs, such as quinine and artemisinin derivatives can effectively clear malaria parasites in blood, however a significant numbers of severe malaria patients including CM patients die or develop severe sequelae regardless of treatment [1], [2]. It is not clear which factors exacerbate mortality among this subset of CM patients, therefore important questions remain to be answered concerning the mechanism(s) of malaria pathogenesis and development of effective therapies. Existing anti-malaria therapy focus on clearance of parasites from blood. This strategy does not prevent secondary deleterious effects such as neurological disorders and cognitive problems caused by parasite derived factors. Recent studies have demonstrated that many pathological changes result from malaria-induced secondary effects which involve various signaling molecules and pathways in the host, indicating that malaria pathogenesis is highly complex and multifactorial, [3], [4], [5].

Recently, it has been shown that increased level of free Heme produced during malaria infection induces inflammation that damages host vascular endothelium which is responsible for induction of fatal cerebral pathogenesis as well as acute lung injury (ALI)/ARDS [4], [5], [6], [7]. Heme oxygenase (HO) is the rate-limiting step enzyme in the degradation of Heme groups to biliverdin, carbon monoxide (CO) and iron. Up-regulation of HO-1 protects against cellular stress including oxidative stress, heavy metal toxicity, UV radiation, and inflammation, thus preventing deleterious effects of Heme and mediating anti-inflammatory and anti-apoptotic functions [8], [9]. HO-1 induced by reactive oxygen species and nitric oxide (NO), has recently been shown to be involved in regulation of angiogenesis [10], [11]. HO-1 may facilitate the repair of injured tissues through inhibition of infiltrating inflammatory cells [12]. Severe malaria is associated with perturbation of inflammatory cytokines, chemokines, anti-inflammatory cytokines and angiopoietic factors [13]. Chemokine CXCL10 is a cytokine belonging to the CXC chemokine family. CXCL10 binds CXCR3 receptor to induce chemotaxis, apoptosis, cell growth and angiostasis [14], [15]. It is expressed early in mice infected with *P. berghei* ANKA (ECM) as well as in human CM [13], [16]. Studies conducted in India and Ghana have identified CXCL10 as a serological marker highly linked with increased risk of fatal *P. falciparum*-mediated CM mortality in humans [3], [17], [18]. Following our report of a role of CXCL10 in fatal human CM, several groups utilized gene knockout mice (CXCL10−/−, CXCR3−/−, IFN-γ−/−) in ECM to confirm and demonstrate the role of CXCL10/CXCR3 interactions in the pathogenesis of fatal CM via the recruitment and activation of pathogenic CD8+ T cells [19], [20], [21]. In fact, survival of mice infected with the lethal strain of *P. berghei* ANKA CM [19] increased to 80% in CXCL10−/− and CXCR3−/− mice when compared with the wild type within the observation period of 10–12 days post infection. In addition to genetic deletion of CXCL10 gene, Nie et al [22] also reported that CXCL10 neutralization with specific antibodies protected against cerebral malaria infection and inflammation. Passive transfer of anti-CXCL10 antibodies reduced the recruitment of inflammatory leukocytes across the blood brain barrier, while genetic deletion of CXCL10 not only alleviated intravascular inflammation but also reduced pRBC sequestration in the brain [22]. Not surprisingly, adoptive transfer of CD8+ cells abrogated protection of CM in CXCR3−/− mice [21]. In addition, NK cells mediate direct cytotoxic activity or reconstitution of capacity of T cells to migrate in response to CXCL10 to the central nervous system (CNS) in CM [23], [24]. Thus, the relationship between over production of Heme, CXCL10/CXCR3, and related signaling mechanisms and fatal CM needs clarification.

The signal transducer and activator of transcription (STAT3) is a signaling molecule which can be activated by pro- and ant-inflammatory stimuli and cellular stresses, therefore STAT3 can be either pro-inflammatory and anti-inflammatory [25]. Many cytokines can activate STAT3, for instance, STAT3 is essential for the function of both interleukin-6 (IL-6) and IL-10, whereas IL-6 is a proinflammatory cytokine, IL-10 is an anti-inflammatory cytokine [26], [27]. Among the cytokines activating STAT3, only IL-10 activates the anti-inflammatory pathway that is STAT3-dependent [28]. Opposing roles of IL-6 and IL-10 mediated by STAT3 might be explained by selective blocking IL-6 and IL-10 signaling by suppressor of cytokine signaling (SOCS3) recruitment, which is part of the STAT3 negative-feedback loop. SOCS3 is a relatively specific inhibitor of gp130 [29], and is a key regulator of IL-6 and IL-10 [26]. SOCS3 selectively blocks signaling by IL-6, specifically prevents activation of STAT3 by IL-6 but not IL-10 [29], thereby preventing its ability to inhibit lipopolysaccharide (LPS)-induced anti-inflammatory signaling. Thus in the absence of SOCS3 in macrophages, the action of IL-6 shifted from inducing a pro-inflammatory responses to a STAT3-mediated anti-inflammatory response [26]. STAT3 protein locates in the cytoplasm in an inactive form and is activated via phosphorylation (pSTAT3) by the Janus tyrosine kinases (JAKs). Although it is known that the proinflammaotry NF-κB pathway [30], [31], Src family kinase [32], [33] and Rho kinase [2] modulate host response to *P. falciparum*, little is known about the role of STAT3 in *Plasmodium* strain infection. The reason that we are interested in STAT3 molecule is that both STAT3 and NF-κB are active during inflammation, the STAT3/NF-κB overlapping sites can be found in the regulatory region of several genes [34]. These transcription factors are suggested to regulate each others' function through competition for overlapping DNA binding sites [34]. Since NF-κB is a well established signaling pathway which contributes to the initiation and development of malaria, we hypothesize that STAT3 may plays an important role in the pathogenesis of severe malaria.

To understand how Heme/HO-1, CXCL10/CXCR3 and STAT3 are involved in the pathogenesis of CM [3], [4], [5], as well as which tissues are involved in the Heme/HO-1 or CXCL10/CXCR3 or STAT3 pathways, and how their expression are regulated in malaria, we conducted a study focus on clarifying the roles of Heme/HO-1, CXCL10/CXCR3 and STAT3 in CM pathogenesis utilizing a well established experimental cerebral malaria (ECM) model, validating results in appropriate *in vitro* methods. We believe that our study will provide a basis for development of novel therapies targeting biological signaling molecules associated with development of fatal malaria. The potential novel therapeutic targets identified in this study will supplement traditional prophylactics and treatments for malaria and improve clinical outcomes while reducing malaria mortality.

## Results

### Infection of C57BL/6 with *P.berghei* ANKA causes brain, lung and kidney damage

**Figure 1:**
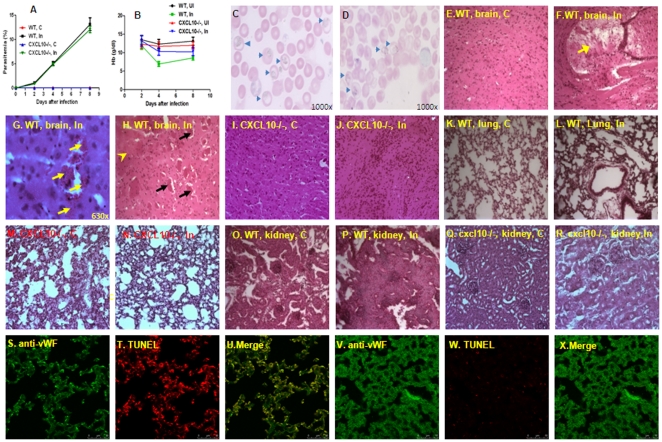
Infection of C57BL/6 with *P.berghei* ANKA causes brain, lung and kidney damage. **1**) C57BL/6 mice were intraperitoneally inoculated with 1×10^6^ *P.berghei* parasitized RBC (pRBC or iRBC) (C = non-infected control, In = infected with PBA), parasitemia and Hb were monitored through mice tail blood samples daily. A: parasitemias in both WT and *CXCL10*−/− mice were below 15% after infection with PBA for 8 days. B: Hb levels were lower in infected WT mice compared to non-infected controls. Hb levels in *CXCL10*−/− mice were similar to corresponding non-infected controls. C: blood smear of infected WT mice with arrow showing parasitized RBC. D: blood smear of infected *CXCL10*−/− mice with arrow showing parasitized RBC. **2**) Paraffin-embedded sections of brain tissues were stained with Hematoxylin-Eosin (HE) (magnification of all panels of Figure 1 is 200× with some exceptions where the magnifications are specifically indicated). Brains of ECM animals showed disruption of vessel walls with endothelial degeneration and cerebral edema as indicated by enlargement of perivascular spaces (Figure 1F). In addition, parenchymal microhaemorrhages (Figure 1G, yellow arrows) were common in white and grey matters in ECM mice. Adherence of pRBC and leucocytes to brain vessels and vascular plugging were present in all sections of ECM mice analysed (Figure 1H, black arrow). Histopathological examination also showed necrotic findings in grey matter in PBA-infected C57BL/6 mice (Figure 1H, yellow arrow head). No histological lesions were detected in non-infected controls of brain tissues (Figure 1E). No obvious pathological lesions were detected in *CXCL10* gene deficient mice (*CXCL10−/−*) infected with PBA (Figure 1J) compared to corresponding controls (Figure 1I). **3**) In the lung of C57BL/6 mice the onset of ECM was characterized by the presence of leucocyte infiltrates and alveolar edema without marked thickening of the alveolar septum by HE staining (Figure 1L) compared to normal control (Figure 1K). 1M and 1N showed HE staining in lung of *CXCL10−/−* mice without and with PBA infection. Apoptotic cells were revealed by TUNEL assay (Figure 1T, in red). The low-power images (Figure 1S) show strong vWF immunoreactivity in pulmonary tissues and blood vessels. Co-localization of vWF-positive and TUNEL-positive cells (Figure 1U) in lung confirmed the presence of apoptotic pulmonary endothelial cells. Figure 1V, 1W and 1X showed no apoptotic pulmonary endothelial cells in non-infected control animals. Kidney histopathology revealed that infection of C57BL/6 with *P.berghei* caused proximal convoluted tubular cell swelling and denaturation. The onset of ECM was characterized by proximal convoluted tubular cell swelling and eosinophilic denaturation as well as narrowed space between tubules (Figure 1P) compared to non-infected control (1O). ). 1Q and 1R showed HE staining in kidney of *CXCL10−/−* mice without and with PBA infection.

C57BL/6 mice were intraperitoneally inoculated with 1×10^6^ *P.berghei* parasitized RBC (pRBC), an inoculum which leads to cerebral malaria in the majority of infected mice. During infection, parasitemia and anemia status were monitored through mice tail blood analysis daily for eight consecutive days. *P.berghei* parasitemias was observed in both wild type (WT) and *CXCL10*−/− mice although below 15% in *CXCL10*−/− mice (Figure 1A). Hemoglobin (Hb) levels was lower in infected wild type mice when compared to *CXCL10*−/− mice as shown in Figure 1B. CXCL10 gene deficient mice had no significant decrease in Hb levels as seen in WT. There was no significant difference in the number of parasitized RBC (arrow) in blood smears of WT (Figure 1C) and *CXCL10*−/− mice (Figure 1D) infected with *P. berghei* (PBA). Figure 1C also showed that similar maturation stages of the parasite was observed to circulate in WT and CXCL10−/− mice. Although *P.berghei* ANKA infected *CXCL10*−/− mice have reduced mortality [19] but have similar level of parasitemia as control mice.

C57BL/6 mice infected with *P.berghei* ANKA is a well accepted murine model of experimental cerebral malaria (ECM) and has been used as a useful surrogate of human CM [4]. This model has been criticized by some researchers [35]. They stated that unlike the pulmonary pathology observed in the murine model by which pRBC sequestration is similar to that observed in human post mortem tissues, encephalopathy in murine model is characterized by monocyte sequestration [35]. However a recent study reported by Baptista et al [36] found that ECM model shares some characteristics of human CM such as intracerebral accumulation of parasitized RBC, which is crucial for the onset of CM pathology. Whether these pRBC are sequestered to host endothelium or not need to be further investigated. Brain histopathological examination showed that infection of C57BL/6 with *P.berghei* caused brain damage. Compared to non-infected controls (Figure 1E, “C” means non-infected control), brains of ECM animals showed significant disruption of vascular endothelial wall, with degeneration and cerebral edema as indicated by enlargement of perivascular spaces (Figure 1F, “In” means PBA infection). In addition, parenchymal microhaemorrhages (Figure 1G, yellow arrows) were common in white and grey matters in ECM mice. Adherence of pRBC and leucocytes to brain vessels and vascular plugging were present in all sections of ECM mice analyzed (Figure 1H, black arrow). Histopathological examination also showed necrotic tissues in grey matter in PBA-infected C57BL/6 mice (Figure 1H, yellow arrow head). No histological lesions were detected in non-infected controls of brain tissues (Figure 1E). No obvious pathological lesions were detected in *CXCL10* gene deficient mice (*CXCL10−/−*) infected with PBA (Figure 1J) compared to corresponding controls (Figure 1I).

The lung tissues in C57BL/6 mice after the onset of ECM were characterized by a discrete presence of leucocytes and alveolar edema without marked thickening of the alveolar septum by HE staining (Figure 1L) compared to normal control (Figure 1K). No obvious pathological lesions were detected in *CXCL10−/−* mice infected with PBA (Figure 1N) as well as non-infected controls (Figure 1M). Apoptotic cells were recognized by a TUNEL assay (Figure 1T, in red) in the lung tissue of WT mice. To determine whether the apoptotic cells are endothelial cells, staining with the endothelial specific anti-vWF antibody (green) was used. The low-power images (Figure 1S) show strong vWF immunoreactivity in pulmonary tissues and blood vessels. Co-localization of vWF-positive and TUNEL-positive cells (Figure 1U) in lung tissues confirm the presence of apoptotic pulmonary endothelial cells. Figure 1V, 1W and 1X showed no apoptotic pulmonary endothelial cells in non-infected control animals.

Kidney histopathological examination indicated that infection of C57BL/6 with *P.berghei* caused proximal convoluted tubular cell swelling and denaturation. The kidney tissues in C57BL/6 after the onset of ECM were characterized by proximal convoluted tubular cell swelling and eosinophilic denaturation as well as narrowed spaces between tubules (Figure 1P) compared to non-infection control (1O). No obvious pathological lesions were detected in *CXCL10−/−* mice infected with PBA (Figure 1R) as well as non-infected controls (Figure 1Q). No apoptotic cells were observed in the glomeruli and tubules (data not shown).

### Heme/HO-1, CXCL10 and STAT3 are involved in *P. berghei* ANKA infected ECM

#### Plasma levels of Heme and HO-1 are increased in *P. berghei* ANKA infected ECM mice

**Figure 2:**
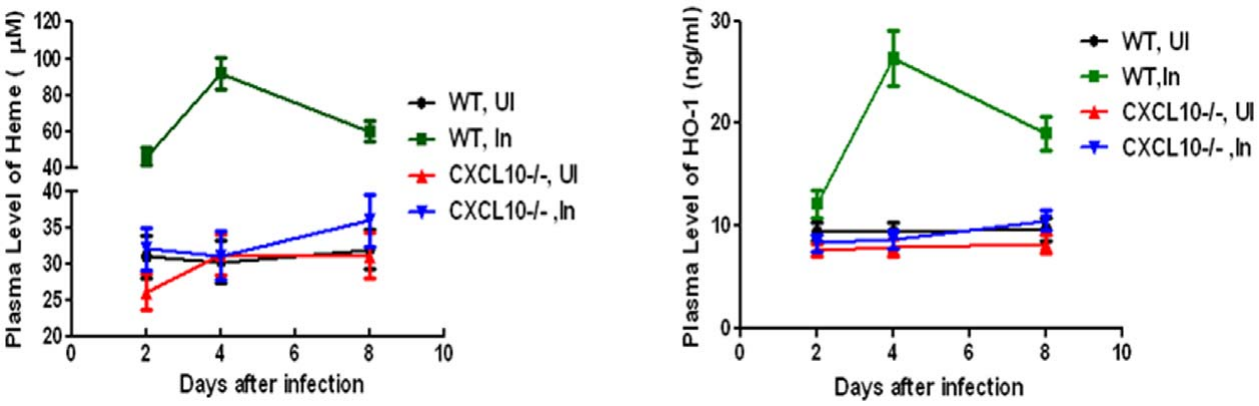
Plasma levels of Heme and HO-1 are increased in ECM mice infected with *P. berghei* ANKA. In left panel of Figure 2, plasma levels of Heme were significantly increased in PBA-infected wild type C57 BL/6 mice (n = 6) than those of non-infected controls (n = 6), indicating that plasma Heme was associated with PBA infection in mice. HO-1 plasma levels were significantly higher in the PBA-infected wild type C57 BL/6 mice compared to controls, suggesting HO-1 may be a protective factor against Heme (right panel). CXCL10−/− infected mice showed the same pattern as CXCL10−/− non-infected mice regarding plasma concentration of Heme and HO-1. When infected with PBA, CXCL10−/− mice do not present the same increase in the levels of Heme or HO-1 as WT mice do during infection (Figure 2). Data are shown as mean ± SD (n = 6 per group).

Plasma levels of Heme significantly increased in infected wild type (WT) C57 BL/6 mice than those of non-infected controls, indicating that plasma Heme is associated with PBA infection in mice (Figure 2, left panel). Additionally, HO-1 plasma levels were significantly higher in the infected wild type C57 BL/6 mice compared to the control mice, suggesting HO-1 could be a protective factor against Heme (Figure 2, right panel). CXCL10−/− infected mice showed the same pattern as CXCL10−/− non-infected mice regarding plasma concentration of Heme and HO-1. When infected with PBA, CXCL10−/− mice do not present the same increase in the levels of Heme or HO-1 as WT mice do during infection (Figure 2).

#### High expression levels of HO-1 and tyrosine-phosphorylated STAT3 (pSTAT3) occur in kidney, brain and lung tissues of mice infected with *P. berghei* ANKA

**Figure 3:**
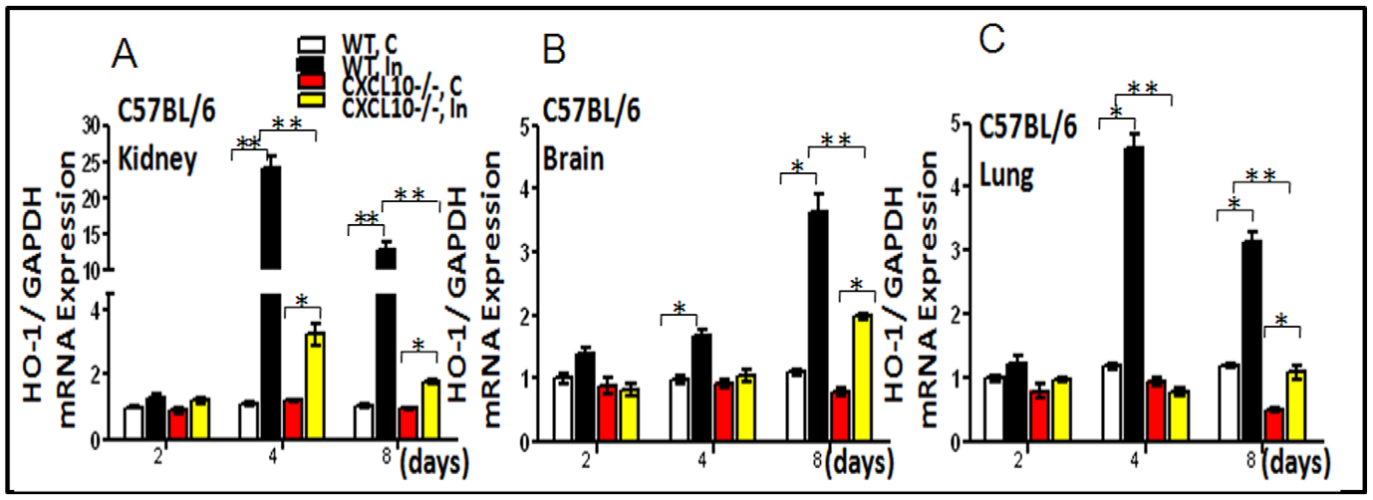
*Hmox1*(HO-1) messenger RNA (mRNA) expression in kidney, brain, and lung as determined by quantitative RT-PCR. Data are shown as mean ± SD (n = 6 per group). *P<0.05; ** P<0.01. mRNA expression of HO-1 in kidney, brain and lung tissues of mice infected with *P. berghei* ANKA are increased significantly. The data showed the mean ratio of HO-1 mRNA expression to GAPDH expression (n = 6) in mice on day 2, 4 and 8 respectively. Infection of C57BL/6 WT or *CXCL10*−/− mice with *P. berghei* (C = non-infected control In = PBA infectetion) up-regulated HO-1 mRNA in kidney (A), brain (B) and lung (C), suggesting HO-1 expression may be protective against *P. berghei* induced damage. Mice deficient in CXCL10 down regulated HO-1 in both uninfected and infected C57BL/6 mice (white vs. red; black vs. yellow), suggesting that transcription of mouse HO-1 gene is positively regulated by CXCL10.

**Figure 4:**
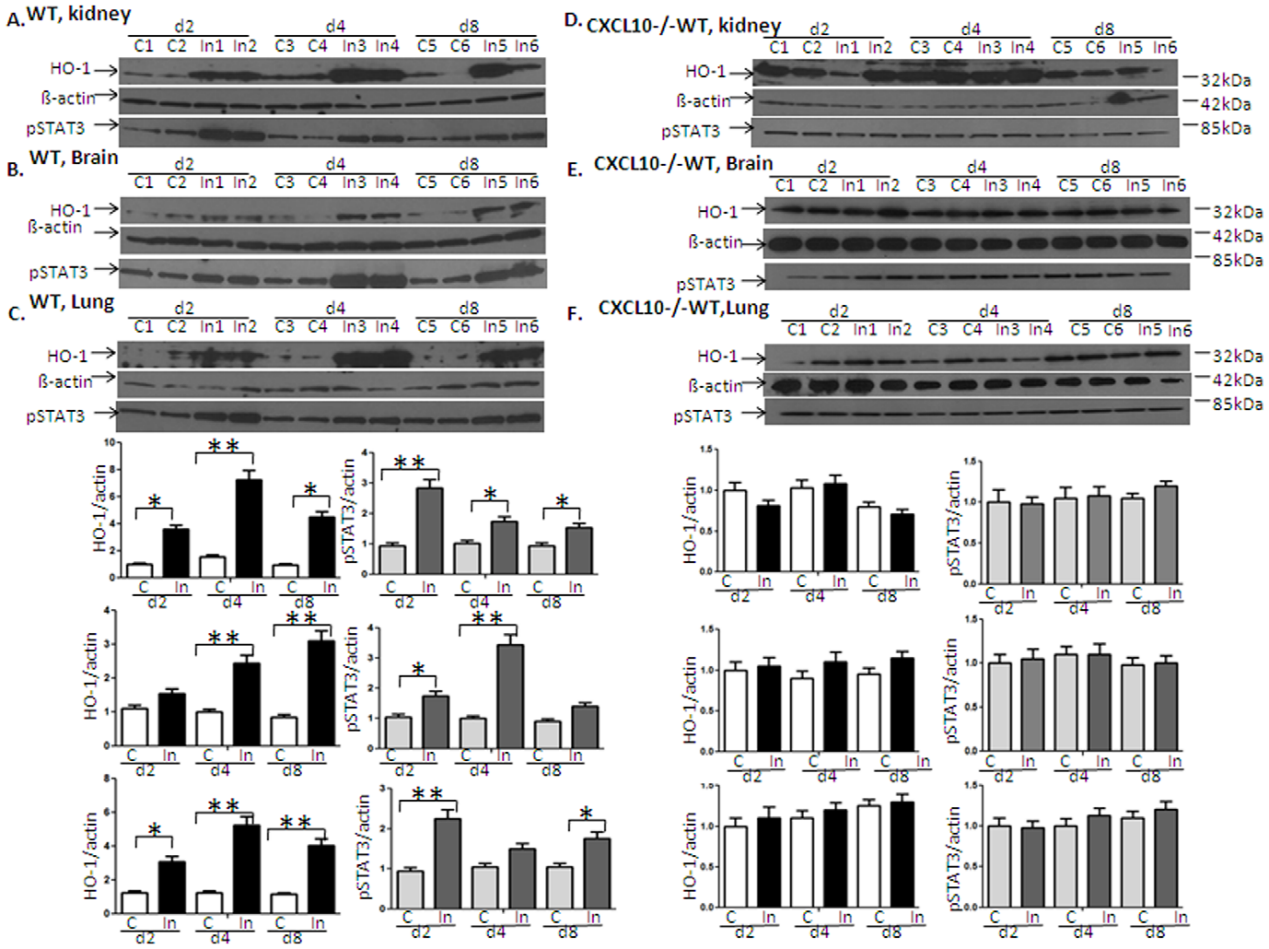
High expression level of HO-1 protein and phosphorylated STAT3 (pSTAT3) occur in kidney, brain and lung tissues of mice infected with *P. berghei* ANKA (n = 6 per group). HO-1 and the active STAT3 molecules-pSTAT3^Tyr705^ protein were examined in kidney (Fig. 4A), brain (Fig. 4B) and lung (Fig. 4C) of some mice by Western blot. *P. berghei* infection up-regulates HO-1, pSTAT3 protein in various tissues of C57BL/6 mice. pSTAT3 level peaked on day 2 in kidney and lung and on day 4 in brain, STAT3 level appeared earlier than those of HO-1, which peaking on day 4 in kidney and lung, on day 8 in brain respectively. However, *P. berghei* infection failed to up-regulate HO-1 protein in *CXCL10−/−* mice (Figure 4D–F). Corresponding densitometric analysis of the bands performed with the ImageQuant program were shown below the Western blot. Data are shown as mean ± SD (n = 6 per group). *P<0.05; ** P<0.01.

**Figure 5:**
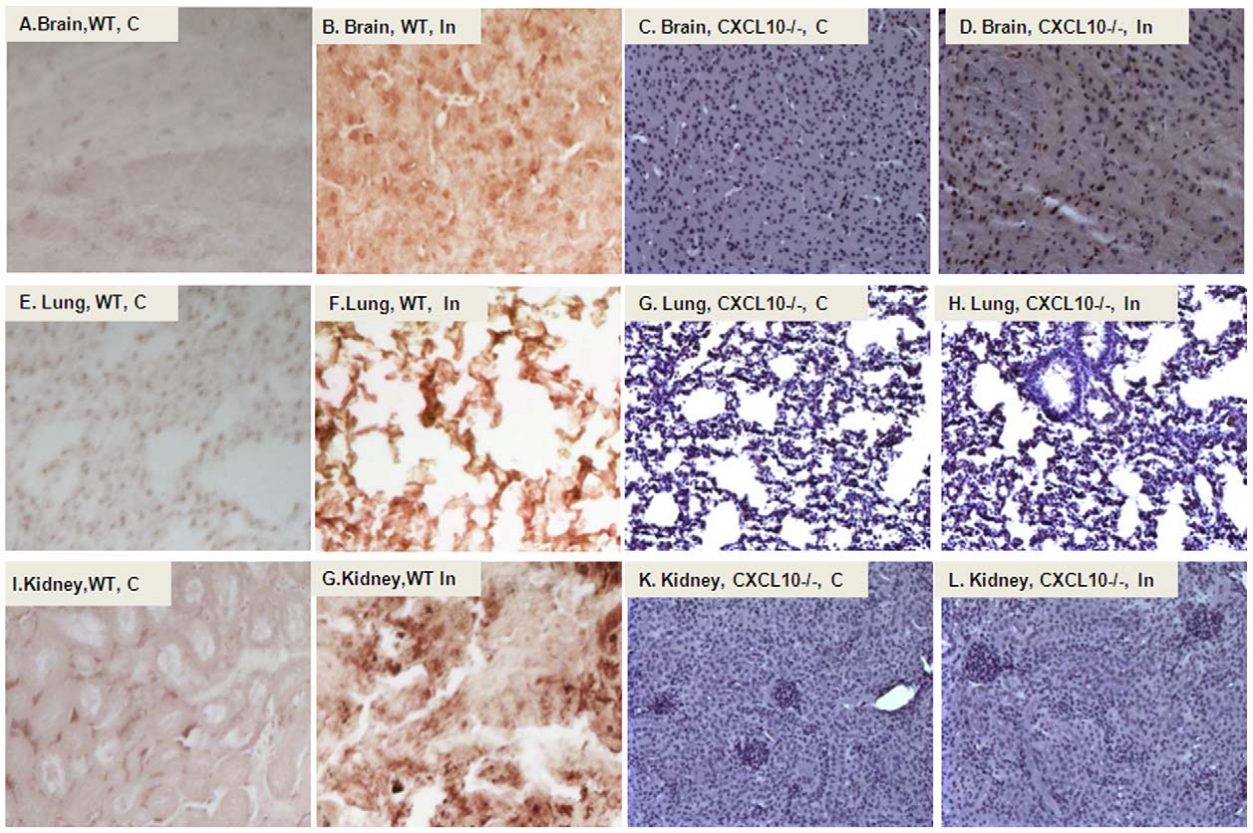
HO-1 protein locates in the nucleus. Tissue sections were deparaffinized followed by rehydration in a standard manner. Specific primary antibody against HO-1 were added at 1∶200 dilution overnight. The activated HO-1 protein was located in the nucleus as showed by the immunohistochemistry assay. HO-1 levels were significantly increased in PBA infected WT of C57 BL/6 mice (Figure 5B vs.5A, 5F vs.5E, 5G vs. 5I), but no significant difference between non-infected and infected group in *CXCL10* gene deficient mice (Figure 5D vs. 5C, 5H vs.5G, 5L vs.5K) was found.

Figure 3 shows the mean ratio of HO-1 mRNA expression to GAPDH expression in mice (n = 6) on day 2, 4 and 8 respectively. Infection of C57BL/6 WT or *CXCL10*−/− mice with *P. berghei* (C = non-infected control, In = infected with PBA) up-regulated HO-1mRNA in the kidney (A), brain (B) and lung (C), suggesting HO-1 expression may be protective against *P. berghei* induced damage in these organs. Mice deficient in CXCL10 down-regulated HO-1 in both uninfected and infected groups (white vs. red; black vs. yellow), suggesting that transcription of mouse HO-1 gene is positively regulated by CXCL10. HO-1 and the active STAT3 molecules-pSTAT3^Tyr705^ protein were also examined in kidney (Figure 4A), brain (Figure 4B) and lung (Figure 4C) by Western blot. Corresponding densitometric analysis of the bands performed with the ImageQuant program (Bio-Rad) were shown below the Western blot. The data demontrated that *P. berghei* infection up-regulates HO-1 and pSTAT3 protein in various tissues of WT mice. pSTAT3 levels peaked on day 2 in kidney and lung and on day 4 in brain but appeared earlier than those of HO-1 which peaked on day 4 in kidney and lung and on day 8 in brain respectively. However, *P. berghei* infection failed to up-regulate HO-1 protein in *CXCL10−/−* mice (Figure 4D–F). HO-1 protein was mostly located in the nucleus as shown by immunohistochemistry (IHC) analysis (Figure 5) [C = non-infected controsl, In = infected with PBA]. Consistent with the levels of mRNA detected by real-time reverse transcription polymerase chain reaction (qRT-PCR) assay as shown in Figure 3 and protein levels detected by Western blot as shown in Figure 4, HO-1 levels were significantly increased in PBA infected WT of C57 BL/6 mice (Figure 5B vs.5A, 5F vs.5E, 5G vs. 5I), but no signicicant difference of HO-1 expression between non-infected and infected group in *CXCL10* gene deficient mice (Figure 5D vs. 5C, 5H vs.5G, 5L vs.5K) were found.

#### High levels of CXCL10 is associated with ECM onset in C57BL/6 mice infected with *P. berghei* ANKA

**Figure 6:**
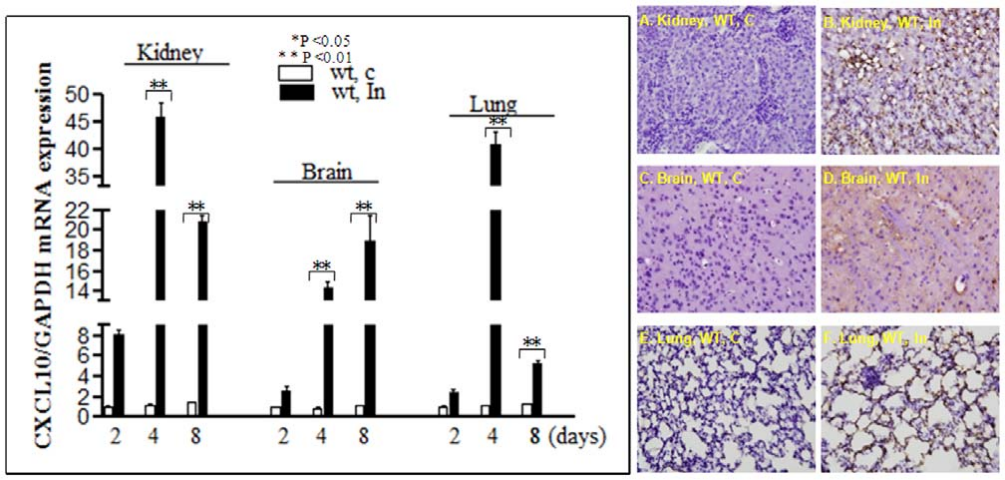
ECM is associated with high levels of *CXCL10* expression in vital organs in C57BL/6 WT mice. Left panel: CXCL10 messenger RNA (mRNA) expression in kidney, brain, and lung as determined by quantitative RT-PCR. Data are shown as mean ± SD (n = 6 per group). *P<0.05; ** P<0.01. ECM onset in C57BL/6 mice infected with *P. berghei* ANKA reveals that levels of CXCL10 mRNA were much higher in kidney, brain and lung tissues in infected than uninfected mice on day 4 and day 8 respectively (left panel). Right panel (Figure 6A–6F): representative examples of HO-1 immunohistochemistry staining in kidney, brain and lung of C57BL/6 wild type mice. Membrane and cytoplasma staining of CXCL10 protein were much stronger in the kidney, brain and lung of PBA infected WT C57 BL/6 mice compared to corresponding controls (Figure 6B vs. 6A, 6D vs. 6C, 6F vs.6E).

Levels of CXCL10 mRNA (Figure 6, left panel) were much higher in kidney, brain and lung tissues in infected than uninfected mice on day 4 and day 8 respectively. The similar results were further confirmed by immunohistochemistry analysis as shown in the right panel of Figure 6. The membrane and cytoplasma staining of CXCL10 protein were much stronger in the kidney, brain and lung of PBA infected WT C57 BL/6 mice compared to corresponding controls (Figure 6B vs. 6A, 6D vs. 6C, 6F vs.6E). These data indicate that fatal ECM is associated with high levels of *CXCL10* expression in vital organs in C57BL/6 WT mice.

### Heme mediated STAT3 activation *in vitro*

#### *HO-1* and *CXCL10* are induced by Heme and its synthetic inducer in mouse endothelial CRL2581 cell line

**Figure 7:**
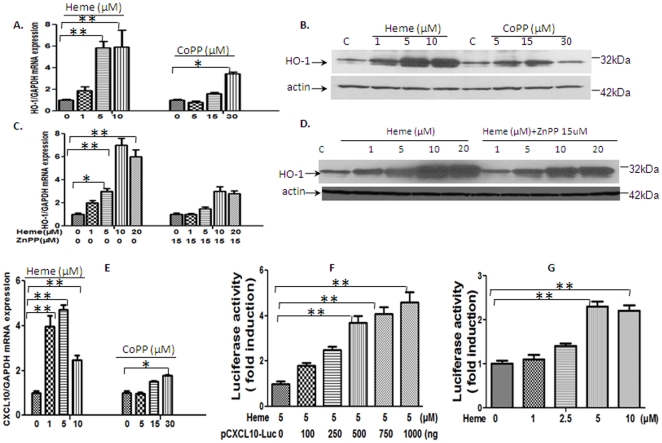
HO-1 and CXCL10 are induced by Heme and its synthetic inducer in mouse endothelial CRL2581 cell line. We exposed mouse brain endothelial cell line CRL-2581 in vitro with Heme at different concentration. The results show that expression of HO-1 mRNA (A) and protein (B) are significantly up-regulated by Heme and HO-1 enzymatic inducer-CoPP treatment. HO-1 expression peaked at 5–10 µM for Heme and 5–15 µM for CoPP. HO-1 mRNA (C) and protein (D) were down-regulated by HO-1 enzymatic activity inhibitor-ZnPP. The expression of CXCL10 mRNA (E) were also significantly up-regulated by Heme and CoPP treatment. The CXCL10 promoter activity is proportional to amounts of CXCL10 luc (F) within the range between 100 ng to 1000 ng when treated with Heme. Figure 7G showed that Heme enhanced the CXCL10 promoter activity in a dose-dependent matter within the range from 1 µM to 5 µM. Data are shown as mean ± SD. *P<0.05; ** P<0.01.

In cerebral malaria pathogenesis, a significant amount of free Heme is produced that induces inflammation that results in damage to host microvascular endothelium. Our studies indicated that transcription of mouse *HO-1* gene is positively regulated by *CXCL10* (Figure 3). STAT3 protein was activated in mice infected with PBA (Figure 4). To determine how Heme, HO-1 and CXCL10 signaling pathways were regulated as well as how STAT3 pathway was associated with these two signaling molecules, mouse endothelial cell line CRL-2581 was treated *in vitro* with Heme to investigate the regulatory mechanism controlling Heme/HO-1, CXCL10 and STAT3 function. The results show that expression of HO-1 mRNA (Figure 7A) and protein (Figure 7B) are significantly up-regulated by Heme and the HO-1 enzymatic inducer-CoPP. HO-1 expression peaked at 5–10 µM for Heme and 5–15 µM for CoPP. HO-1 mRNA (Figure 7C) and protein (Figure 7D) were down-regulated by HO-1 inhibitor ZnPP. The expression of CXCL10 mRNA (Figure 7E) was also significantly up-regulated by Heme and CoPP treatment. The CXCL10 promoter activity was proportional to amounts of CXCL10 luc (Figure 7F) within the 100 ng to 1000 ng range when treated with Heme. Figure 7G shows that Heme enhances the CXCL10 promoter activity in a dose-dependent manner within a 1 µM to 5 µM range.

#### STAT3 activation is induced by Heme

**Figure 8:**
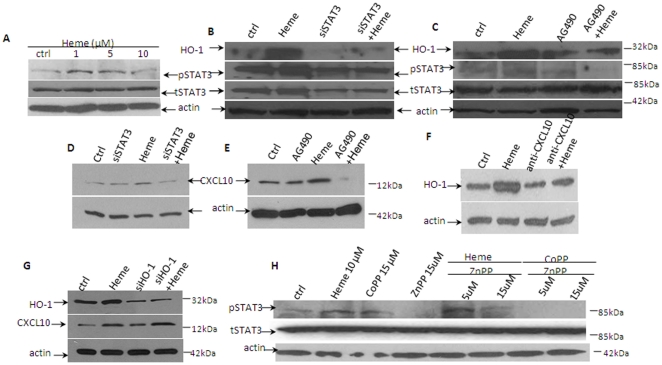
STAT3 activation is induced by Heme in CRL-2581 cells. Decreased activation of STAT3 by siRNA-STAT3 (siSTAT3) and AG490 down regulates HO-1 and CXCL10 protein. pSTAT3 was increased by Heme from 1 µM and peaked at 5 µM and then decreased (A). When pSTAT3 was reduced by siSTAT3 (B) or pharmacological inhibitor of Jak, AG490 (C), HO-1 protein expression was inhibited correspondingly. These data indicated that Heme induced production of HO-1 by way of STAT3 signaling pathway. Reduced activation of STAT3 caused by siSTAT3 (Figure 8D) and AG490 (Figure 8E) also caused reduced expression of CXCL10. We futher found when CXCL10 was blocked by anti-CXCL10 antibody, HO-1 induction by Heme was decreased to one half, indicating that CXCL10 directly induce HO-1 expression (Figure 8F). Reduced HO-1 expression by siHO-1 increased CXCL10 expression which implies that HO-1 also modulates CXCL10 expression (Figure 8G). Interestingly, pSTAT3 level was increased by CoPP and decreased by ZnPP (Figure 8H), which indicates that HO-1 also regulates STAT3 signaling.

**Figure 9:**
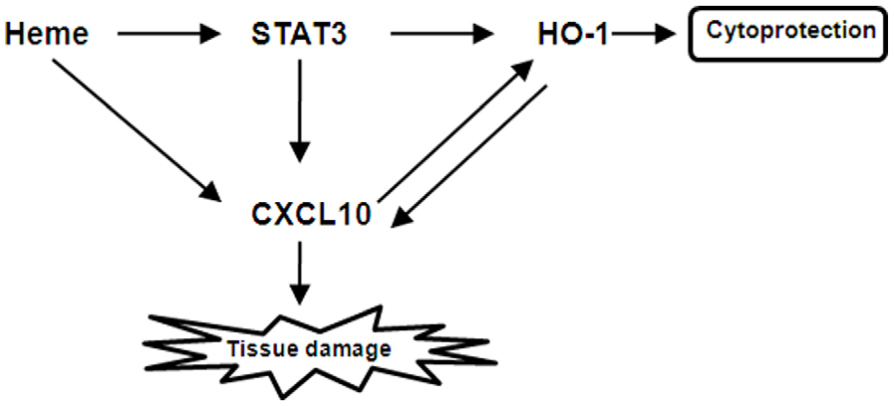
Schematic model for the regulation of Heme/HO-1, CXCL10 and STAT3. 1) Heme induced HO-1 and CXCL10 production, 2) Heme induced HO-1 and CXCL10 expression through STAT3 activation, 3) A mutual signaling regulation loop exists between HO-1 and CXCL10.

Decreased activation of STAT3 by siRNA-STAT3 (siSTAT3) and AG490 down regulates HO-1 and CXCL10 protein. Recently Chen's group [37] found that the lethal strain of *P. yoelli* (XL) caused STAT3 activation which inhibited host protective immunity and resulted in high parasitemia and death. To analyze the involvement of STAT3 during the course of *P. berghei* ANKA infection, a process which produces significant amount of free Heme [4], we assessed the expression levels of total STAT3 (tSTAT3) and pSTAT3 in CRL-2581 cells. pSTAT3 was increased by Heme concentrations from 1 µM to 5 µM and then decreased (Figure 8A). When pSTAT3 is reduced by siSTAT3 (Figure 8B) or by a pharmacological inhibitor of Jak, AG490 (Figure 8C), HO-1 protein expression was inhibited. Reduced activation of STAT3 caused by siSTAT3 (Figure 8D) and AG490 (Figure 8E) also caused reduced expression of CXCL10. These data indicate that Heme induces the production of HO-1 or CXCL10 via a STAT3 signaling pathway. We futher found when CXCL10 was blocked by anti-CXCL10 antibody, HO-1 induction by Heme was decreased to one half, indicating that CXCL10 directly induce HO-1 expression (Figure 8F). Reduced HO-1 expression by siHO-1 increased CXCL10 expression which implies that HO-1 also modulates CXCL10 expression (Figure 8G). Interestingly, pSTAT3 level was increased by CoPP and decreased by ZnPP (Figure 8H), which indicates that HO-1 also regulates STAT3 signaling. None of the treatment had effects on tSTAT3. Based on our findings, a schematic model for the regulation of Heme/HO-1, CXCL10 and STAT3 was shown in Figure 9.

## Discussion

Severe malaria pathogenesis is associated with dysfunction of multiple organs [38]. The fatal cases are composed of cerebral malaria (CM) and other severe forms of malaria such as severe malaria anemia (SMA). Accumulating evidence indicates that acute lung injury (ALI)/ ARDS caused by malaria is responsible for significant proportion of the mortalities in children and pregnant woman [6], [39]. Additionally, acute renal failure is a complication of *Plasmodium* infection in non-immune and semi-immune African children which often results in mortalities [40], [41]. However, the precise mechanism(s) responsible for fatal CM-induced brain damage and malaria-induced pulmonary disruption are unclear. Two main hypotheses have been proposed for human CM. The first is the mechanical hypothesis, which proposes that infected Red Blood Cells (iRBC or pRBC) binding to endothelial cells (EC), obstruct blood flow in micro capillaries leading to low tissue perfusion, compromised oxygenation and tissue damage. The second is the immunopathological hypothesis which proposes that host hyper-inflammatory responses to parasite infections responsible for eliminating *P. falciparum* parasites cause edema, dysfunction in blood brain barrier (BBB), organ failure and death. However, the role of malaria-induced secondary effects involving various signaling molecules of the host is unkown. Our current study has demonstrated that (1) infection of C57 BL/6 with *P.berghei* ANKA (PBA) causes significant tissue damage, (2) Heme/HO-1 and CXCL10 are involved in the pathogenesis of CM and that the level of free Heme correlates with PBA infection in mice, (3) Expression of HO-1 in tissues may be protective against Heme and PBA induced damage, (4) High levels of CXCL10 is associated with ECM onset in PBA infected mice, (5) STAT3 is activated by PBA infection *in vivo* and Heme *in vitro*, (6) Heme up-regulates HO-1 and CXCL10 production trough STAT3 pathway, and regulates CXCL10 at the transcriptional level *in vitro*, (7) HO-1 transcription is positively regulated by CXCL10, (8) HO-1 regulates STAT3 signaling. Taken together, our data indicate that Heme/HO-1, CXCL10/CXCR3 and STAT3 molecules as well as related signaling pathways play very important roles in inflammation and organ damage in the pathogenesis of severe malaria.

Heme and HO-1 interaction moved center stage in cerebral malaria research in 2007, when Mota's group reported that HO-1 and carbon monoxide (CO) suppress the pathogenesis of ECM [4]. HO-1 was then found to be capable of inhibiting vascular occlusion in transgenic sickle cell mice (sickle cell disease is a hemolytic disease) [42], [43]. From these studies it seems that the ability of individuals to respond to increase in Heme by producing HO-1 may be a crucial endogenous protective factor. However, some other studies have refuted the findings that HO-1 protects the development of CM [44]. These studies suggests that the frequency of short (GT)n alleles (<28 repeats), which may lead to high level of HO-1, is markedly higher in CM patients [44]. Moreover, liver stages of the *Plasmodium* was markedly reduced in *Hmox1−/−* mice [4]. These conflicting results suggest that the regulated expression of HO-1 is quite complex in different tissues at different stages of the *Plasmodium* life cycle. Therefore, further experimental and epidemiological studies are necessary to unveil the role of Heme and HO-1 interactions in severity of malaria. HO-1 is a heat shock protein (hsp32), which is an integral membrane protein of the smooth endoplasmic reticulum [45], and is the only inducible isoform of HO. The expression of HO-1 occurs at low levels in most tissues under physiological conditions [40]. HO-1 can localize to distinct subcellular compartments. Inducible HO activity appeared in plasma membrane, cytosol, mitochondria [46], isolated caveolae and nucleus [47] in cell culture models. Early studies indicate that HO-1 in mitochondria and caveolae performs important biological and physiological actions [46], although the function of HO-1 in caveolae and nucleus is not completely understood. The nuclear form of HO-1 serves potentially as a transcriptional regulator [46]. Under conditions of hypoxia, hemin or Heme-hemopexin, HO-1 translocates to the nucleus. Nuclear translocation compromises the HO activity, but nuclear localization of HO-1 protein functions to up-regulate genes that promote cytoprotection against oxidative stress [45]. Our data showed that levels of HO-1 were significantly increased in plasma and tissues (both mRNA and protein), the activated HO-1 protein was mostly located in the nucleus, which supports the hypothesis that HO-1 protects against Heme and tissue damage. In *CXCL10*−/− mice, PBA infection caused modest increase in HO-1 mRNA (Figure 3), but not in HO-1 protein [(neither in plasma (Figure 2) nor tissue (Figure 4)], there could be a number of reasons. HO-1 protein may be expressed but at levels below detectable limits, or may be rapidly degraded. As protein expression reflects functional adaption observed in species phenotype [48], HO-1 in either case probably did not exert the expected protection. Considering the fact that there was no significant difference in free Heme level between *CXC10*−/− infected mice and non-infected controls (Figure 2), we postulated that HO-1 activation may not be required under this situation.

Animal models provide valuable biological information under controlled circumstances. However, different mice strains show variations in susceptibility to rodent malaria [49], [50], this may reflect qualitative or quantitative differences in host immune response to the parasite and differences in the pathogenicity of sub-strains of murine malaria parasite species. C57BL/6 infected with PBA shares many features similar to human CM. However, lung damage might not be severe enough to cause animal death [6]. This may explain why the pathological manifestation in lung and kidney was modest our study.

Our observation of Hb levels being lower in infected wild type mice is consistent with previous studies which showed that *P.berghei* ANKA infection in C57BL/6 results in anemia. CXCL10 gene deficiency prevents decrease in Hb levels (Figure 2). Since the level of free Heme is not increased (Figure 2), it is possible that this may occur through reduction of hemolysis of infected RBC. But the compromised clearance of uninfected RBCs or erythroid response could not be excluded as a possibility [51], [52], [53].

A recent study in Ghanaian patients demonstrated an association between fatal CM and increased serum and cerebrospinal fluid (CSF) levels of proinflammatory and proapoptotic factors including CXCL10, IL-1ra, sTNFR1, sTNFR2 and sFas and decreased serum and CSF levels of neuroprotective angiogenic growth factors (PDGFb) [3]. Further investigation in Indian patients confirmed findings from Ghana, thus indicating that CXCL10, sTNFR2 and sFas are positively correlated, while angiogenic and anti-apoptotic factors, VEGF is negatively correlated with mortality associated with CM [17]. Studies from a murine CM model also confirmed importance of CXCL10/CXCR3 interactions in the pathogenesis of fatal CM through the recruitment and activation of pathogenic CD8+ T cells [20], [54]. CXCL10−/− and CXCR3−/− mice are partially resistant to *P. berghei*-mediated CM [19]. The animal studies demonstrate that high level of CXCL10 in tissues is associated with ECM in PBA infected mice, which is consistent with previous reports relating to human studies. Our studies to determine the mechanisms by which CXCL10 is up-regulated using *in vitro* cell culture models revealed that Heme regulates CXCL10 at the transcriptional level *in vitro*.

Our results also suggest that CXCL10 is positively associated with HO-1 gene expression, and may be involved in the regulation of HO-1 (Fig. 3 and Fig. 8F). Interestingly, an emerging body of evidence demonstrates that HO-1 gene also regulates CXCL10 expression. For instance, HO-1-mediated cytoprotection is mediated by suppression of CXCL10 during liver ischemia and reperfusion injury [55], [56], [57] and kidney transplantation [58]. Our results indicating that reduced HO-1 expression by siHO-1 increased CXCL10 expression (Figure 8G) support these previous findings. Additionally, HO-1 may enforce angiostatic action via CXCL10 during renal injury [12]. This observation supports the views that a mutual signaling regulation loop exists between HO-1 and CXCL10. Detailed understanding of the characteristic signaling abnormalities could contribute to novel approaches in diagnosis and treatment of severe malaria.

STAT3 can be activated by pro- and ant-inflammatory stimuli and cellular stresses, therefore STAT3 can be either pro-inflammatory and anti-inflammatory [25] depending on the recruitment of SOCS3, which is part of the STAT3 negative-feedback loop. In the absence of SOCS3 in macrophages, the action of a STAT3-mediated IL-6 shifted from inducing a pro-inflammatory responses to an anti-inflammatory response [26]. The active form of STAT3 is quickly translocated to the host cell nucleus. pSTAT3 was reported to be a potent negative modulator of the Th1-mediated inflammatory response. It is also an activator of a variety of genes which are important for immune modulation [59], [60]. Chen's group reported that lethal *Plasmodium yoelii* (*P.yoelii*, XL) induced activation of STAT3 in the early phase of infection, the dominant pSTAT3 response may dampen the development of protective immunity which results in high parasitemia and death [37]. In the present study, we determined that STAT3 is activated during PBA infection *in vivo* and Heme *in vitro*. Heme activated STAT3 works as a pro- inflammatory factor. Heme induced up-regulation of HO-1 and CXCL10 is through the STAT3 pathway. Our results also indicate that the activation of STAT3 precedes the peak levels of HO-1 induced by PBA infection, which is consistent with previous report [37]. Interestingly our results also show that HO-1 regulates STAT3 signaling in cell culture model. The Heme/HO-1, CXCL10 and STAT3-related signaling involved in CM pathogenesis are highly complex. Thus, a full understanding of the interactions of these pathways will facilitate the development of novel strategies for intervention against malaria.

The mortality associated with severe malaria (CM, SMA and ALI/ARDS) remains high in spite of availability of adequate treatment [61]. Most adjunct treatments have failed to improve the outcome of severe malaria since these treatments focus mainly on the clearance of parasites. Recent studies have demonstrated that many severe pathological changes result from malaria induced secondary effects involving signaling molecules of the host [3], [4], [5]. The signaling pathways activated by *Plasmodium* infection and cross talks between them are still poorly understood and definitely represent a fruitful area for further study.

## Methods

### Ethics statement

This study was carried out in strict accordance with the recommendations in the Guide for the Care and Use of Laboratory Animals of the National Institutes of Health. The protocol was approved by the Institutional Animal Care and Usage Committee (IACUC) of Morehouse School of Medicine (Permit Number 09-06).

### Mice

6–8-week-old female C57BL/6 and C57BL/6 *CXCL10*−/− mice (The Jackson Laboratory, Bar Harbor, Maine) were used as a CM strain for the experiments. The mice were housed under standard conditions in the animal facility of Morehouse School of Medicine (MSM) and supplied with food and water *ad libitum*. The animals were allowed to adjust to their new environment for at least 3 days before the testing. Mice were infected intraperitoneally with 1×10^6^ *P.berghei* parasitised erythrocytes (pRBC) in 0.2 ml phosphate buffered saline (PBS), which were obtained from whole blood of a mouse infected with frozen stocks of PBA [62].

Non-infected C57BL/6 and C57BL/6 *CXCL10−*/− mice injected with normal RBC served as control groups. Mice were sacrificed at days 2, 4 and 8 to harvest brain, kidney and lung for analysis of different purposes. All animal studies conformed to national regulations on animal experimentation and welfare, and the Care of Laboratory Animal Resources (CLAR) guidelines for the care and use of laboratory animals.

### Cell culture

CRL-2581, a murine endothelial cell line obtained from American Type Culture Collection (Manassas, VA), were maintained and grown in DMEM medium with 10% heat-inactivated fetal bovine serum (FBS) (Life Technologies, Inc.), and penicillin/streptomycin (Hyclone). Cells were maintained at 37°C under 5% CO2.

### Antibodies and reagents

Polyclonal antibody against HO-1 was obtained from Assay Designs (Ann Arbor, MI). STAT3 and phospho-STAT3 were purchased from Cell Signaling Technology. Antibody to β-actin was obtained from Sigma-Aldrich. Polyclonal anti-vWF antibody was purchased from DAKO (Carpinteria, CA). Anti-mouse CXCL10 antibody (Clone: 134013, Ig Class: rat IgG_2A_) was purchased from R&D System, Inc. All secondary antibodies used for Western blot were purchased from Calbiochem. AG490 (a JAK2 inhibitor) was obtained from Calbiochem. STAT3 siRNA, HO-1 siRNA and control siRNA were purchased from Santa Cruz. The CXCL10 promoter-luciferase construct was obtained as a generous gift from Narayan Bhat (Department of Neuroscience, Medical University of South Carolina, Charleston, SC). Hemin, CoPP and ZnPP were purchased from Frontier Scientific (Logan, UT).

### Measurement of parasitemia and hemoglobin (Hb)

Parasitaemia and anemia status were monitored daily by thin blood smears of tail blood and were tested for eight consecutive days. Parasitemia was determined by counting the number of pRBC in a total count of 1000 RBC in thin blood smears fixed by methanol and stained by Giemsa (Sigma, St. Louis, MO). Anemia was evaluated by counting the Hb by HESKA CBC Diff Veterinary Hematology System.

### Quantification of Heme

Plasma was centrifuged for 5 min at 4°C at 1100 g to remove contaminating red blood cells and were passed through a Microcon YM-3 column (Millipore) for 60 min at 14°C at 21,000 g to remove proteins (MW>3 kDa). Free Heme was quantified in protein-depleted plasma using a chromogenic assay according to the manufacturer's instructions (BioAssay System, Hayward, CA).

### Enzyme immunoassay (EIA)

EIA was performed using mouse HO-1 immunoset kit from Assay Designs (Ann Arbor, MI). A 96-well-plate (Nunc, Roskillde, Denmark) was coated with 100–200 ng of mouse HO-1 capture antibody in phosphate buffered solution (PBS) and allowed to incubate overnight at 4°C. The plates were then blocked by addition of 1% bovine serum albumin (BSA) in PBS for at least one hour at room temperature. Coated wells were washed with 0.1% Tween-20 in PBS, recombinant HO-1 standard and samples were loaded into the wells in triplicate incubating at room temperature for 1 hour. After 3 times of washing, HO-1 detection antibody was made in PBS with 1% BSA and added into each well and incubated at 37°C for 1 hours. Anti-mouse immunoglobulin (Assay Designs, Ann Arbor, MI) conjugated to horseradish peroxidase (HRP) was incubated in wells and colorimetric development was performed by addition of the HRP substrate. Absorbance was read at 450 nm.

### Western blotting

Animal tissues were homogenized and incubated for 60 min at 4°C in lysis buffer, samples were separated by SDS/PAGE, and separated proteins were transferred to nitrocellulose membranes and identified by immunoblotting. Primary antibodies were obtained from commercial sources, these antibodies were diluted at the ratio of 1∶1000 according to manufacture's instruction, while secondary antibodies included HRP-conjugated anti-rabbit and anti-mouse antibodies were obtained from Calbiochem. Blots were developed with Supersignal Pico or Femto substrate (Pierce). A densitomeric analysis of the bands were performed with the ImageQuant program (Bio-Rad).

### Immunohistochemistry (IHC) and Immunofluorescence staining

Animal organs were removed after intracardial perfusion with PBS, sectioned (5 µm) and stained for H&E. Sections were deparaffinized with xylene followed by rehydration through a graded series of ethanol and double distilled water in a standard manner. Specific primary antibodies were added at 1∶200 dilution overnight. The sections were then incubated with biotinylated secondary antibodies (1∶200, Vector, Burlingame, CA, USA) for 60 min at RT, the avidin-biotin complex (ABC Kit, Vector, Burlingame, CA, USA) for 30 min. For TUNEL assay, the *in situ* cell death detection kit (TMR red; Boehringer-Mannheim, Mannheim, Germany) were used. The sections were incubated with the TUNEL reaction solution for 60 min at 37°C in the dark.

For labeling of endothelial cells with vWF antibody, sections were incubated with a rabbit polyclonal anti-vWF antibody (1∶250, DAKO, Carpinteria, CA) for 60 min at 37°C. The sections were then incubated with biotinylated goat anti-rabbit IgG (1∶200, Vector, Burlingame, CA), and in avidin-biotin complex (ABC Kit) for 30 min. Fluorescein staining was developed using the Alexa-488 fluorescence system (Molecular Probes, Carlsbad, CA). Fluorescent images was collected by using a Zeiss LSM510 confocal microscope and images were captured with LSM software version 2.3 (Carl Zeiss, Wetzlar, Germany).

### Real-time RT-PCR analysis

Animal tissues or cell pellets were stored in Trizol reagent and homogenized in fresh Trizol. Total RNA were isolated from cells using an RNeasy Mini Kit (Qiagen, Valencia, CA). cDNA were synthesized from the isolated RNA using iScript cDNA Synthesis Kit (Bio-Rad Laboratories, Inc.). Reverse transcription was performed by using random hexamers at 25°C for 5 minutes, 42°C for 30 minutes, and 85°C for 5 minutes. Quantitative PCR were performed using iQ SYBR Green Supermix (Bio-Rad Laboratories, Inc.) in a CFX96 Real-Time PCR System machine (Bio-Rad Laboratories, Inc.). The data was analyzed using CFX96 Real-Time PCR System (Bio-Rad Laboratories, Inc.). Primer sequences for the mouse *HO-1*, *CXCL10* and *GAPDH* genes were described in Table 1.

**Table 1:** Primers for real-time polymerase chain reaction.

Primer set	Forward primer (5′→3′)	Reverse primer (5′→3′)
Mouse HO-1	GCCACCAAGGAGGTACACAT	CTTCCAGGGCCGTGTAGATA
Mouse CXCL10	AAGTGCTGCCGTCATTTTCT	CCTATGGCCCTCATTCTCAC
Mouse GAPDH	AACGACCCCTTCATTGAC	TCCACGACATACTCAGCAC

### Luciferase reporter gene assay

CRL-2581 cells were transfected using lipofectamine 2000 (Invitrogen) with 0.75 µg of CXCL10 promoter-luciferase construct together with 100 µg of pRL-TK, a cytomegalovirus-Renilla vector to control transfection efficiency. The amount of total DNA transfected was equalized with the appropriate amounts of control vectors. After transfection at different indicated points, cells were harvested and lysed in reporter lysis buffer (Promega, Madison, WI). Luciferase activity was determined by using the Dual Luciferase Kit (Promega) and a luminometer (Turner Design, Sunnyvale, CA) according to the manufacturer's recommendation. All luciferase results were normalized to Renilla activity from the co-transfected pRL-TK plasmid. The data for luciferase activity was expressed as fold induction with respect to control cells and was the mean ± standard error of triplicate samples.

### Statistical analysis

The results obtained in this work were from triplicate experiments performed independently by identical methods. EIA data and densitometeric measurements from Western blot analyses in mice were log-transformed to normalize the distribution for infected (n = 6) and control (n = 6) samples. Data were expressed as the mean ± standard error of mean (SEM). Data from the *P. berghei* ANKA infected and control groups were compared. The p values were determined by using nonparametric Mann-Whitney U-test. A value of p<0.05 was considered statistically significant.
